# Managing thyroid dysfunction in selected special situations

**DOI:** 10.1186/1756-6614-6-2

**Published:** 2013-02-04

**Authors:** Durr e Sabih, Mohammad Inayatullah

**Affiliations:** 1 Multan Institute of Nuclear Medicine and Radiotherapy, Nishtar Hospital, Multan 60000, Pakistan; 2 Department of Medicine, Nishtar Medical College and Hospital, Multan 60000, Pakistan

## Abstract

Managing thyroid dysfunction is simple at first glance, the idea is to bring hormone levels in the euthyroid range, treat with antithyroid drugs, radio-iodine or surgery if toxic and replace with thyroxine or T3 if hypothyroid. Complexities arise when there are coexisting conditions that affect the thyroid or are affected by thyroid dysfunction and this review will deal with the special situations that need care when correcting thyroid hormone levels.

## Pregnancy

Pregnancy induces several changes in thyroid function. An early change is the reduction of the available iodide pool due to an increase of iodine clearance by the kidneys and the transplacental transfer of iodide and iodothyronine to the fetus. To avoid iodine deficiency, increase in dietary iodine intake is recommended for pregnant women (250 μg/d vs. 150 μg/d in non-pregnant women [1]).

Thyroid related problems are common in pregnancy. Obvious hypothyroidism can occur in up to 2% pregnancies and subclinical hypothyroidism in up to 3% of pregnant women, hyperthyroidism can occur in up to 0.4% pregnancies [2]. Post partum thyroiditis can be seen in up to 10%. Comparison with normal prevalence rates can be difficult because thyroid dysfunction tends to increase with age, however in one study 1.5% females under 30 were hypothyroid [3], in another study prevalence of previously undiagnosed thyrotoxicosis was 0.47% [4].

Most thyroid dysfunction is autoimmune in nature and Graves’ disease accounts for thyrotoxicosis and Hashimoto’s thyroiditis for hypothyroidism in most cases.

While de novo thyroid problems are common in pregnancy; autoimmune thyroid disease, specially Graves’ disease, might get better during pregnancy and the disease can show post partum exacerbation [5,6].

The evaluation of thyroid dysfunction and treatment both are complicated by the fact that:

1. There is an increase in thyroid binding globulin level in early pregnancy to almost twice the non-pregnant levels secondary to reduced clearance of TBG by the liver and an increase in its production under the influence of estrogen. There is increased total T3 and T4 levels [7,8]. Free T4 (FT4) and Free T3 (FT3) levels are not affected by increased serum thyroglobulin concentrations but caution needs to be exercised in the interpretation of FT4 levels and laboratories should establish their own trimester specific reference range [9]. First and third trimester FT4 levels are significantly different [10].

2. Human chorionic gonadotrophin (hCG) produced by the placenta, has a weak thyrotropin (TSH) like activity and causes a transient increase in FT4 concentration during the first trimester. TSH levels tend to be low throughout pregnancy but especially so in the first trimester, so this alone cannot be used as a marker for thyroid functional status [11]. The upper normal limits of TSH in the first trimester have recently been revised to 2.5 mIU/L [12]. In twins the TSH values are even lower.

The best tests are the combination of TSH and FT4, TSH being interpreted according to trimester specific values for the population (Table 1). There are some recommendations that include thyroid peroxidase antibodies (TPOAb) in the thyroid workup of pregnant women.

**Table 1:** **Trimester specific levels for TSH (from ref **[12]**)**

	
First Trimester	0.1-2.5 Miu/L
Second Trimester	0.2-3.0 Miu/L
Third Trimester	0.3-3.0 Miu/L

It is important to recognize and treat thyroid dysfunction in pregnancy because of a number of adverse affects of both hypo and hyperthyroidism for both the mother and fetus (Table 2).

**Table 2:** Adverse effects of thyroid dysfunction in pregnancy

		
Maternal effects	Hypothyroidism	Microcytic anemia
		Preeclampsia
		Placental Abruption
		Post partum Hemorrhage
		Cardiac dysfunction
		Miscarriage
		Spontaneous abortion
		Preterm labour
		Low birth weight
	Hyperthyroidism	Hyperemesis gravidarum
		Miscarriage
		Infection
		Preeclampsia
		Preterm delivery
		Congestive cardiac failure
		Thyroid storm
		Placental abruption
Fetal effects	Hypothyroidism	Prematurity
		Low birth weight
		Congenital anomalies
		Stillbirth
		Poor, delayed neurological development*
	Hyperthyroidism	Prematurity
		Small for age
		Intrauterine death
		Fetal (neonatal) goiter
		Fetal (neonatal) hypothyroidism

* The fetus needs transplacental supply of thyroid hormones during the first trimester for early brain development.

### Hypothyroidism in pregnancy

The lab diagnosis of hypothyroidism rests on the demonstration of increased trimester specific TSH levels in the pregnant woman, the distinction between overt hypothyroidism (OH) and Subclinical hypothyroidism is further made depending upon the presence of low or normal FT4 levels when interpreted in the context of trimester specific reference range [10].

Women with overt or subclinical hypothyroidism should be treated with oral thyroxine to normalize the TSH within the trimester specific reference range. Women with pre-existing hypothyroidism should undergo frequent monitoring (monthly till 20 weeks, then again at around 32 weeks) of TSH and the dose of thyroxine adjusted to maintain this within the trimester specific reference range. But they must be instructed to immediately increase their thyroxine dosage by 25% upon missing a period. Some women will need up to 80% more thyroxine to maintain their TSH levels within the normal reference range but fine adjustment can be done at the 6 week mark.

After delivery a previously hypothyroid woman should go back to her pre-pregnancy dose but this should be adjusted by getting a TSH done 6 weeks after delivery. Up to half will need an upward dose adjustment from the pre-pregnancy level.

### Thyrotoxicosis

Up to 1% women may develop thyrotoxicosis in pregnancy. Commonest autoimmune cause is Graves’ disease [13]. Some women might experience transient “gestational hyperthyroidism” in the first half of pregnancy. This presents with increased FT4 and suppressed TSH levels without the presence of serum markers of thyroid autoimmunity [14]. Gestational hyperthyroidism may be related to elevated hCG levels and has been associated with hyperemesis gravidarum. The presence of nodularity should increase the suspicion of probable T3 toxicosis and the test protocol should then include T3 levels too.

Gestational hyperthyroidism, once confirmed (absence of maternal goiter, eye signs, antibodies) should be managed conservatively, the aim being the treatment of hyperemesis with fluids and medication to control vomiting. The use of antithyroid medication is not recommended because the hormone levels become normal by about 20 weeks of gestational. In situations where the distinction between Graves’ disease and Gestational hyperthyroidism cannot be made, it might be prudent to start low dose antithyroid therapy, adjusting the dose, or discontinuing these in light of follow-up TSH levels.

Once it has been decided that the patient needs antithyroid treatment, propylthiouracil (PTU) should be used in the first trimester; after which treatment should be switched to Methimazole or carbimazole. Methimazole or Carbimazole in the first trimester can cause “MMI or carbimazole embryopathy” [15-18], propylthiouracil is safe in this period but can be hepatotoxic at any time [19] so the risks should be explained to the patient. Generally low initial doses of antithyroid drugs are recommended and suggested doses are PTU, 50-300 mg/day, Carbimazole 10-15 mg/day, MMI 5-15 mg/day [12]. Beta blockers like propranolol can be used to control severe symptoms initially but should be discontinued within 2–6 weeks, long term beta blockers has been associated with growth retardation, fetal bradycardia and a higher rate of spontaneous abortion.

The goal of treatment is to maintain FT4 levels at or just above the upper limit of normal range adjusting the dose after every 2–6 weeks in the light of FT4 assays. A corollary to this recommendation is that subclinical thyrotoxicosis needs no treatment. Generally, combination of antithyroid drugs and thyroxine (block and replacement therapy) is not recommended.

## Cardiovascular disease

No peripheral deiodination of T4 occurs in the heart muscle and the myocyte relies entirely on circulating T3 for its actions. Thyroid hormone effects include reduction in systemic vascular resistance, increased resting heart rate, left ventricular contractility and blood volume, it also increases erythropoietin production and red cell mass [20].

Thyroid dysfunction has significant cardiac effect. Even when treated and euthyroid, patients with thyroid dysfunction are more prone to cardiovascular disease [21]. Mild and subclinical thyroid dysfunction is associated with an increased mortality in patients with cardiovascular disease [22-24].

### Thyrotoxicosis

Many features of thyrotoxicosis derive from the effect of excess thyroid hormones on the heart and include palpitation, tachycardia, exercise intolerance (from an inability to further increase heart rate), wide pulse pressure and sometimes atrial fibrillation. Cardiac output might increase by 50-300%.

*Angina*, with ECG changes of ischemia, can be provoked by thyrotoxicosis, even in the presence of normal coronary arteries, this is presumed to be due to coronary artery spasm or a prothrombotic state induced by thyrotoxicosis in young patients [25]. In older patients with preexisting or suspected cardiac ischemia, this might reflect an increased oxygen demand by the myocardium. [26,27]. Treating thyrotoxicosis with available options improves cardiac symptoms.

#### Atrial fibrillation

Almost all patients with thyrotoxicosis have sinus tachycardia but the most significant tachyarrythmia is atrial fibrillation that may be present in 2-20% of patients [28]. Although thyrotoxicosis is responsible for only 1% of all atrial fibrillations, thyroid testing in all patients with new onset AF is justified because of the ability to restore the rhythm to normal with reversion of the patient to euthyroid state.

The ventricular rate slows when patients of thyrotoxicosis induced atrial fibrillation are treated with β-blockers. The thyroid status should be corrected with antithyroid drugs or radio-iodine.

#### Heart failure

Thyrotoxic patients may exhibit features of heart failure. It is a precipitating factor and tachycardia and atrial fibrillation may cause rate limited left ventricular dysfunction and failure [29]. The definitive treatment of choice of the thyrotoxicosis in these patients is radioiodine [30,31].

### Hypothyroidism

Just like thyrotoxicosis, the cardiovascular manifestations of hypothyroidism are prominent. These include bradycardia, mild hypertension, narrow pulse pressure, cold extremities, increased systemic vascular resistance, decreased cardiac contractility and output, accelerated atherosclerosis and coronary artery disease, cold intolerance and fatigue [20].

ECG changes include a prolongation of the QT interval that predisposes to ventricular irritability [32]. There is prolongation of the isovolumic diastolic relaxation. Many patients can develop pericardial or pleural effusion.

Managing hypothyroidism in the presence of cardiac disease in young adults is easy and a full replacement dose can be initiated. Some cases of chest pain, particularly the elderly will remain challenging and thyroid replacement therapy might exacerbate angina [33]. Treatment should be started with a very low dose (25–50 μg/day) with a very slow increase (every 6–8 weeks); this will usually improve cardiac function [34].

#### Low T3 syndrome

Up to a third of patients with heart failure have low T3 levels with normal T4 and TSH levels [35]. In this setting a reduced T3 is a strong predictor of mortality. There might be a place for physiological T3 replacement in such cases [29]. Placebo controlled trials suggest benefits of short term T3 replacement in neuroendocrine profile, stroke volume and cardiac output without increase in cardiac workload [36].

## Drugs

### Amiodarone

Amiodarone is a popular drug used for cardiac rhythm disturbance (both atrial and ventricular). It inhibits peripheral deiodination of T4 and decreases T3 concentration in the serum. It has a very high iodine content and can cause either hypothyroidism (5-25% of treated patients) or thyrotoxicosis (20-10% of treated patients) [37].

#### Hypothyroidism

Patients on amiodarone therapy should be regularly tested for thyroid function by TSH and replacement therapy should be started if the TSH levels increase. Amiodarone is not discontinued and the replacement therapy is optimized by monthly TSH tests. High TSH and a mid range or high normal T4 level would suggest subclinical hypothyroidism and should be treated with thyroxine replacement therapy [38].

#### Thyrotoxicosis

Two types of amiodarone induced thyrotoxicosis have been described, type 1 occurring in those with pre-existing thyroid disease and goiter, and type 2 occurring in normal sized thyroids with no previous history of thyroid disease. Type 2 thyrotoxicosis is a destructive thyroiditis and in many cases the patient passes from a thyrotoxic state to a hypothyroid state in a few months. Type 2 thyrotoxicosis can occur suddenly at any time during treatment and thyroid function tests should be done at the slightest suspicion of a possible thyroid dysfunction.

Treatment of amiodarone induced thyrotoxicosis is difficult and some times the distinction of type 1 from type 2 is difficult or cannot be made. No consensus exists on the most effective and appropriate regimen. Discontinuation of amiodarone rarely helps, perhaps because the terminal half life is very long (about 4 months) [38]. β- blockers and steroids (unless the patient is a diabetic) have been used. After cessation of amiodarone, high doses of thiourea derivatives like carbimazole have been suggested but no consistent benefits have been demonstrated. In patients with pre-existing Graves’ disease or toxic multinodular goiter, radioiodine can be given (type 1), but it is not useful in type 2 amiodarone thyrotoxicosis.

In some patients, amiodarone cannot be discontinued due to risks of exacerbation of thyrotoxicosis and serious ventricular arrhythmias upon cessation [39,40] and other reasons. It might be safer in these to continue with amiodarone and treat thyrotoxicosis aggressively although this tends to reduce the chances of a successful outcome [38]. Thyroidectomy is an option in such a situation [41] and has been associated with improvement in cardiac as well as thyroid function. Thyroidectomy has been done under local anesthesia in patients deemed unfit for general anesthesia [42]. Plasmapheresis has occasionally been used but with inconsistent success [43]. Table 3 describes the therapeutic options in different types of amiodarone induced toxicosis.

**Table 3:** **Treatment of Amiodarone induced thyrotoxicosis (from ref **[38]**)**

	**Type 1 thyrotoxicosis**	**Type 2 thyrotoxicosis**
Amiodarone *can* be discontinued	Carbimazole, 40-60 mg/day	Withdrawal will suffice in many cases
	Propylthiouracil 100-150 mg four times a day	Steroids may needed for up to 3 months
	Potassium perchlorate 0.5 g twice a day	
	Radioiodine	
Amodiarone *cannot* be discontinued	Antithyroid drugs	Surgery
	Radio-iodine	Plasmapheresis
	Surgery	

### Lithium

Lithium is a psychoactive drug and is used in depression. It is concentrated by the thyroid and impairs thyroidal iodine uptake, it also inhibits iodotyrosine coupling and inhibits thyroid hormone secretion [44]. It leads to hypothyroidism in 20% and goiter in up to 40% patients; [45]. Rarely it may cause thyrotoxicosis due to thyroiditis or Graves’ disease [46]. Hypothyriodism should be treated by thyroxine replacement and lithium therapy should be continued. Similarly, thyrotoxicosis should be treated with the available therapeutic options without discontinuing lithium.

### Interferon

Interferon related hypothyroidism was first reported in 1985 [47]. These patients probably have a genetic predisposition to autoimmune thyroid disease [48]. The prevalence of thyroid disease in patients treated with interferon varies from 1 to 35% in different reports [49,50]. This might reflect different methodologies used to diagnose thyroid disease or variation in individual susceptibility to thyroid disease with interferon treatment. It has also been argued that viral infection, specially by HCV and to a lesser extent hepatitis B may predispose to the development of thyroid autoimmune disease [51], but this has not been conclusively demonstrated [52]. The commonest thyroid finding in patients treated with interferon is the presence of thyroid related antibodies with normal thyroid function; however, thyroiditis, Graves’ disease and hypothyroidism can all occur, sometimes in the same patient, destructive thyroiditis and thyrotoxicosis are additional problems secondary to inflammation of the thyroid [53-55].

Discontinuation of interferon is rarely indicated but may become necessary in severe destructive thyroiditis and Graves’ disease.

Patients who become hypothyroid during interferon therapy should have replacement therapy. Their TSH levels should continue to be monitored after completing therapy because the hypothyroidism might reverse after discontinuing interferon [56].

Those who develop thyroiditis can be treated with steroids.

Destructive thyrotoxicosis may be treated with β - blockers and steroids can be added if symptoms are uncontrolled; interferon may need to be withdrawn, although this probably does not influence the natural history of the disease [54].

Those with mild Grave’s disease, should have continued interferon therapy with concomitant low dose antithyroid medication. Severe Graves’ disease is unlikely to resolve needing very high doses of antithyroid drugs raising concerns about their intrinsic hepatotoxicity. Interferon might need to be discontinued, awaiting effect of definitive therapy with radio iodine or surgery; after which interferon may be restarted.

A case of panhypopituitarism has been reported [57]. Such cases need to be treated with steroids preceding thyroxine replacement (see section on panhypopituitarism).

## Diabetes

Many studies have demonstrated a higher prevalence of thyroid disease in diabetics (13.4%), that have been as high as 31.4% for female type-1 diabetes [58]. The same paper suggests that diabetics be screened annually for thyroid function. The majority have hypothyroidism (Subclinical 50%, overt 40%), while thyrotoxicosis and thyroiditis are less common [24]. Common susceptibility genes have been identified that may offer insight into the higher association of both conditions.

***Thyrotoxicosis*** promotes hyperglycemia. There is increased degradation of insulin and the release of biologically inactive insulin precursors [59]. There is increased glucose absorption from the intestine [60] and increased hepatic glucose output. There is also increased lipolysis and an increase in free fatty acids that stimulate hepatic gloconeogenesis. In short, there is worsening of glycemic control in diabetics and in some, thyrotoxicosis can be the cause of diabetic ketoacidosis [61]. While managing thyrotoxicosis the concurrent effects on blood glucose levels should be kept in mind and the hypoglycemic agents or insulin doses optimized, as the need for these reduces with better thyroid control.

***Hypothyroidism*** causes reduced rates of glucose production by the liver, leading to decreased insulin requirement. Recurrent hypoglycemic episodes can be the first sign of early hypothyroidism in a known diabetic patient [62]. Patients with subclinical hypothyroidism and diabetes are more prone to nephropathy and retinopathy, favouring the screening of diabetics for thyroid status.

Metformin has been implicated in reducing TSH levels in a small group of patients. This is an isolated effect that is not associated with any changes in FT3 or FT4 levels [63].

The goal of thyroid replacement in these patients is to achieve a euthyroid status with a TSH of between 0.3 and 3.0 mIU/ml [64].

## Asthma

### Thyrotoxicosis

An association between worsening of asthma and thyrotoxicosis has been known for several decades [65,66]. Despite this known association the routine use of thyroid function tests in all asthmatics has not been recommended [67] but there may be a place for thyroid investigation in persons with worsening asthma. A euthyroid status has a positive effect on asthma control [68]. In one series all asthmatic patients developed more serious symptoms as their thyrotoxicosis became more obvious [69], all improved when they became euthyroid. Therapy of asthma in these cases becomes difficult because the thyrotoxic state induces and aggravates the symptoms associated with β agonist therapy, and the anti tachycardia treatment of thyrotoxic symptoms with nonspecific β blockers exacerbates asthma. ^131^I has been given for early radical treatment of thyrotoxicosis with good results [70].

### Hypothyroidism

Hypothyroidism improves coexisting asthma. Correction of hypothyroidism causes worsening of symptoms and difficulties in controlling asthma [71]. There is blunting of response to bronchodilator therapy with an inverse relationship between levels of thyroid function and airway beta adrenergic responsiveness [72]. Caution must be used when correcting hypothyroidism in asthmatics because of the risk of exacerbating asthma.

## Addison’s disease

Increased TSH levels, with normal or reduced T4 levels are often found [73]. Clinical symptoms of tiredness and an elevated TSH with low T4 will prompt a diagnosis of hypothyroidism and prescription of thyroxine as replacement therapy. Thyroxine replacement alone in these patients is dangerous and may precipitate an adrenal crisis [74,75]. Corticosteroid therapy will often correct the thyroid biochemical imbalance too [76]. If thyroxine replacement is deemed necessary it should follow steroid replacement.

Screening all patients of hypothyroidism for adrenal deficiency is not recommended [77], but those patients who are suspected to be hypothyroid because of raised TSH and get worse after thyroxine therapy should be reevaluated clinically as well as biochemically for a possible coexisting adrenal insufficiency.

## Hypopituitarism (secondary hypothyroidism)

Given the relationship between the pituitary and target organ hormones, pituitary hormones are good indicators of target organ function and in a converse manner target organ hormones area good indicators of pituitary function. A raised TSH suggests impaired thyroid function, a low TSH increased thyroid function; a low T4 levels along with normal or low TSH suggest reduced pituitary function.

The patient may be asymptomatic or suffer an acute collapse.

Symptoms vary according to the predominant hormone deficiency and the rapidity of onset [78]. Hypothyroidism was the second commonest presentation following hypogonadism in one series [79].

Symptoms are usually similar to those of primary target organ deficiency. Symptoms of thyrotropin deficiency are the same as primary hypothyroidism. T4 is low but TSH does not show the expected increase that one expects in primary hypothyroidism and might be low or in the normal range. Failure of increase in TSH following thyrotopin releasing hormone would be confirmatory but is rarely needed to make the correct diagnosis.

The goal of treatment is hormone replacement but an extremely important caveat is to ensure that either the glucocorticoid secretion is normal or to precede thyroxine replacement by glucocorticoid therapy. This will avoid an adrenal crisis as the metabolism of glucocorticoids increases under the influence of thyroxine.

## Competing interests

The authors declare that they have no competing interests.

## Authors’ contributions

Author DS designed the contents of the paper, carried out the literature search, drafted the manuscript and is willing to take public responsibility for the article. Author MIU helped designed the contents of the paper, carried out the literature search, undertook critical review and editing of the manuscript and is willing to take public responsibility for the article. All authors read and approved the final manuscript.
